# The prevalence, pathophysiology, and treatment of fecal incontinence in patients with Crohn’s disease: a systematic review and meta-analysis

**DOI:** 10.3389/fmed.2025.1590971

**Published:** 2025-05-27

**Authors:** Yuting Shi, Yujia Zhu, Yixin Lan, Li Xu

**Affiliations:** ^1^The First Clinical Medical College of Zhejiang Chinese Medical University, Hangzhou, Zhejiang, China; ^2^Ningbo Hospital of Traditional Chinese Medicine, Zhejiang Chinese Medical University, Ningbo, Zhejiang, China; ^3^Department of Anorectal Surgery, The First Affiliated Hospital of Zhejiang Chinese Medical University, Hangzhou, Zhejiang, China

**Keywords:** fecal incontinence, Crohn’s disease, prevalence, systematic review, meta-analysis

## Abstract

**Background:**

Fecal incontinence (FI) is a common complication in patients with Crohn’s disease (CD), but there is a relative lack of comprehensive information on its prevalence, pathophysiology, and treatment interventions. This study aims to systematically evaluate the prevalence, pathophysiology, and treatment interventions of FI in patients with CD, providing a reliable dataset for clinical reference.

**Methods:**

As of October 2024, articles were identified through a comprehensive search of PubMed, Web of Science, The Cochrane Library, Embase, and Scopus databases. The review included literature on the prevalence, pathophysiology, and treatment interventions for FI in patients with CD. Using the Stata 14.0 software package, the prevalence of FI among patients with CD was estimated with a random-effects model. Heterogeneity was assessed by calculating the I^2^ statistic and reporting the *p*-value from the chi-squared test for heterogeneity. Sensitivity analysis was performed to evaluate the robustness of the pooled effect estimate. Subgroup analysis and meta-regression were conducted based on various study characteristics, such as study design, sample size, and geographic region, to identify potential sources of heterogeneity. Publication bias was assessed using Egger’s test. Due to the inability to pool data across studies, risk factors, pathophysiology, and treatment interventions were described qualitatively.

**Results:**

In this analysis, a total of 25 studies were included. Fifteen of these studies assessed the prevalence of FI among 7232 patients with CD, yielding a pooled estimated prevalence of 34.8% (95% CI: 24.0%–46.5%). Six studies investigated the pathophysiology, suggesting that FI may be associated with decreased anal resting pressure, rectal compliance, and altered rectal sensation. Five studies evaluated potential treatment interventions, indicating that neuromodulation therapies such as posterior tibial nerve stimulation and sacral nerve stimulation may be effective for FI. Anti-tumor necrosis factor therapy in conjunction with surgical interventions may improve FI. Furthermore, pelvic floor behavioral treatment may improve FI and enhance quality of life when pharmacological treatments are ineffective.

**Conclusion:**

This study provides insights into the prevalence, pathophysiology, and treatment interventions of FI among patients with CD. The findings indicate that the prevalence of FI in CD patients is 34.8%. Further research is necessary to gain a deeper understanding of the pathophysiology of FI and to develop effective management and treatment interventions.

**Systematic review registration:**

https://www.crd.york.ac.uk/PROSPERO/displayy _record.php?ID=CRD42024583028, identifier CRD42024583028.

## 1 Introduction

Crohn’s disease (CD) is a chronic inflammatory disease of the gastrointestinal tract with symptoms evolving in a relapsing and remitting manner. It is also a progressive disease that leads to bowel damage and disability (1). The incidence and prevalence of CD varies across geographic regions, with the highest epidemiological burden in Europe, Oceania and North America (2). In North America, reported incidence rates of CD range from 6.30 to 23.82 per 100,000 person-years, and in Eastern Asia from 0.06 to 3.32 per 100,000 person-years (3). CD is now global, in part due to rising incidence rates of adult and pediatric disease in middle-income countries (2, 4).

Fecal incontinence (FI) is defined as the involuntary loss of solid or liquid stool in individuals aged ≥4 years and is one of the most burdensome symptoms reported by patients with inflammatory bowel disease (IBD) (5). In 2013, a random sample of 10,000 members from the British national Crohn’s & Colitis UK organization was invited to participate in a study (6). Among the respondents, 74% reported experiencing FI at least occasionally. The study found a wide range of estimated prevalence, varying from 25 to 75%. Similarly, a 1991 study involving 108 IBD patients reported a prevalence of 29% for FI (7). Despite being a common symptom, FI often goes unreported, as both clinicians and patients tend to focus more on other clinical manifestations closely associated with CD, such as abdominal pain, diarrhea, perianal issues, and extraintestinal symptoms. As a result, doctors rarely screen for FI, and patients do not typically volunteer this symptom, even though it can severely impact quality of life (8). Consequently, the true prevalence and impact of FI in CD may be underestimated, underscoring the need for a more comprehensive evaluation.

The pathophysiology of FI in patients with CD is multifactorial. Research indicates that CD-related inflammation can lead to structural damage of the internal and external anal sphincters, reducing sphincter pressure and compromising the ability to maintain continence (9). Rectal compliance is often impaired due to chronic inflammation, fibrosis, or surgical treatment, limiting the rectum’s capacity to accommodate stool and resulting in an increased likelihood of urgency and leakage (10). Altered rectal sensation is another critical abnormality, with patients frequently reporting both heightened urgency and diminished sensation of stool presence, further increasing the risk of FI (11). Reports evaluating anorectal motility and its association with FI are contentious, with few studies including small sample sizes. Taken together, these factors highlight the complexity of FI in CD and emphasize the need for targeted diagnostic and therapeutic strategies.

The management of FI typically involves a range of conservative therapies, including dietary modifications, pharmacological treatments, pelvic floor rehabilitation, and behavioral interventions (12). However, while these approaches are widely studied and implemented in the general population, the management of FI in patients with CD presents unique challenges. The unpredictability of disease flare-ups, the extent of perianal involvement, and the frequent need for surgical interventions make the management of FI in this population particularly complex. The most effective treatment available is colostomy, which is not usually accepted by most patients (13). Moreover, while novel therapeutic modalities such as stem cell therapy and biologic treatments have been explored in the management of perianal CD (14, 15), their role in the specific management of FI remains under-explored. Thus, targeted research is critical to developing effective, evidence-based management strategies for this challenging condition.

In summary, FI as a complication of CD requires further investigation, from its prevalence and pathophysiology to the effectiveness of treatment interventions. Therefore, this study systematically evaluated and developed targeted management strategies to improve patient care.

## 2 Methods

### 2.1 Protocol and registration

The authors conducted a systematic review and meta-analysis in accordance with the Preferred Reporting Items for Systematic Reviews and Meta-Analyzes (PRISMA) guidelines (16). The study was reported following both the PRISMA and AMSTAR (Assessing the Methodological Quality of Systematic Reviews) guidelines (17). The study protocol has been registered with PROSPERO (CRD42024583028).

### 2.2 Search strategy

Two authors independently selected relevant articles from PubMed, Web of Science, The Cochrane Library, EMBASE, and Scopus electronic databases, covering the period from inception to October 14, 2024. Detailed search strategies for each database are provided in the Supplementary material.

### 2.3 Selection criteria

Inclusion criteria: (1) Adult patients with a confirmed diagnosis of CD; (2) Studies explicitly report the presence of FI and define or diagnose it; (3) Cohort studies and cross-sectional studies reporting the prevalence and risk factors of FI in CD patients were used to assess these aspects, while any observational or experimental studies reporting the pathophysiology and treatments were included to evaluate the underlying mechanisms and treatment options; (4) Studies written and published in English.

Exclusion criteria: (1) Review, case report, letter, comment, or conference abstract; (2) Unable to obtain the full data or text.; (3) Studies that do not include CD patients or fail to report separate data for CD patients; (4) Studies that do not report outcomes related to FI or provide incomplete data; (5) Duplicate publication.

### 2.4 Data extraction

Two researchers independently performed literature searches and imported the references into EndNote for individual screening and management. Initially, titles and abstracts were reviewed for preliminary screening, followed by the selection of eligible studies based on inclusion and exclusion criteria, ultimately determining the final studies to be included. In the event of a disagreement between the two researchers, a third researcher was responsible for resolving the conflict. Researchers used a standardized form to extract basic characteristics of the studies, including the first author, year, country, study type, sample size, sample source, method to assess disease activity, diagnostic criteria for FI, number of individuals with FI, and quality assessment.

### 2.5 Quality assessment

Two reviewers independently assessed the quality of the selected studies. The JBI Critical Appraisal Checklist for Studies Reporting Prevalence Data is utilized to appraise all prevalence studies (18). The risk of bias for cross-sectional studies was evaluated using the Agency for Healthcare Research and Quality (AHRQ) tool (19), with scores ranging from 0 to 3 indicating low quality, 4 to 7 indicating medium quality, and 8 to 11 indicating high quality. For cohort studies, the Newcastle–Ottawa Scale (NOS) (20) was used to assess methodological quality. The NOS includes eight items across three domains, with scores of 0–4, 5–6, and ≥7 corresponding to low, medium, and high quality, respectively. The Joanna Briggs Institute (JBI) critical appraisal tool (21) was applied to evaluate case series studies, categorizing them as low quality (0–3 points), medium quality (4–7 points), or high quality (8–11 points). The risk of bias in each randomized controlled trial (RCT) was evaluated according to the Cochrane Risk of Bias 2.0 framework (22), categorizing the risk of bias as low, some concerns, or high based on the predefined criteria. Any discrepancies between the two reviewers were resolved through discussion, with a third author consulted when necessary.

### 2.6 Data analysis

Statistical analysis was conducted using Stata 14.0 software. For the prevalence, a random-effects model was used to pool the proportion of CD patients with FI. The aggregated prevalence and corresponding 95% CI were reported. Heterogeneity was quantified using the I^2^ statistic (23). I^2^ values were categorized as mild (<25%), moderate (25%–50%), severe (50%–75%), and highly severe (>75%) heterogeneity (24). The findings are illustrated in the form of forest plots. Subgroup analyses were performed to explore the sources of heterogeneity in the meta-analysis. Sensitivity analyses using a leave-one-out approach evaluated the robustness of pooled estimates. In addition, Egger’s test was applied to investigate publication bias (25). Due to the inability to pool data from different studies, the risk factors, pathophysiology, and treatment interventions were described qualitatively.

Pre-specified subgroup analyses were initially planned to explore heterogeneity across age, gender, and geographical regions. However, due to incomplete reporting of these variables in the included studies, the following alternative subgroups were analyzed based on available data: (1) Sample source (online vs. offline); (2) Study design (cross-sectional vs. cohort studies); (3) Diagnostic criteria (patient report vs. professional instrument). Online data sources were defined as studies collecting cases through web-based surveys or social media platforms, while offline sources included hospital or clinic-based recruitment.

## 3 Results

**FIGURE 1:**
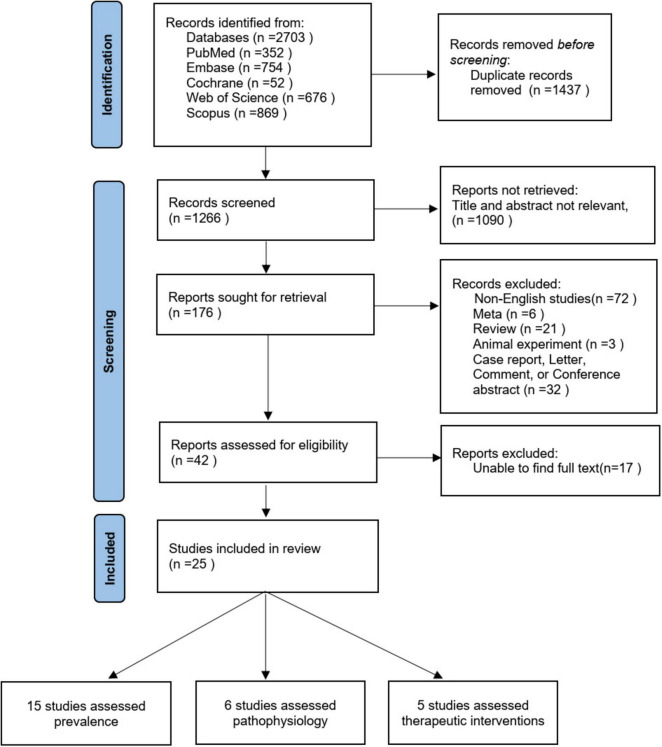
Flowchart for selection of included studies. One study evaluated both prevalence and pathophysiology.

The initial search identified a total of 2703 studies, of which 1437 were duplicates. After the review of titles and abstracts, 1090 of those articles were excluded, leaving 176 validated studies. Of these 176 articles, 25 were determined to meet all inclusion criteria after full text review and quality assessment, of which 15 (11, 26–39) evaluated the prevalence of FI, 6 (37, 40–44) investigated pathophysiology, and 5 (45–49) assessed potential treatments (one article evaluated both prevalence and pathophysiology). The flowchart of study selection process is presented in Figure 1.

### 3.1 Prevalence

#### 3.1.1 Studies characteristics

Of the 15 studies, 11 (11, 29–38) employed a cross-sectional design, and 4 (26–28, 39) utilized a cohort design, involving 7,232 patients with CD. The majority of studies were published in the past 5 years and conducted in the USA (5), the Netherlands (2), the UK (1), France (2), China (1), Israel (1), Japan (1), Germany (1), and Brazil (1). The results are summarized in Table 1.

**TABLE 1:** Summary of studies describing prevalence of FI in CD patients.

Study	Country	Study type	Subjects	Source of subject recruitment	Method to assess disease activity	Diagnostic criteria for FI	No. (%) with FI	Quality assessment
Michelassi, 2000	USA	Cohort study	224 CD	A surgical department	Endoscopy	Patient report	11 (5)	7
Mueller, 2007	Germany	Cohort study	97 CD	An outpatient department	N/A	Patient report	12 (12)	7
Brochard, 2017	France	Cohort study	173 CD	A tertiary gastroenterology center	HBI	Patient report; CCIS	65 (38)	9
Vollebregt, 2017	The Netherlands	Cross-sectional study	325 CD	A medical center	Fecal calprotectin	Patient report; St. Marks incontinence score, CCIS	65 (20)	9
Kochar, 2018	USA	Cross-sectional study	2378 CD	National online survey	sCDAI	Patient report; GI-PROMIS	380 (16)	9
Vollebregt, 2018	The Netherlands	Cross-sectional study	529 CD	A Dutch Crohn’s and Colitis patients’ organization	HBI; PDAI	Patient report; St. Marks incontinence score	306 (58)	7
Dibley, 2021	UK	Cross-sectional study	740 CD	Six hospitals	HBI	Patient report	445 (60)	7
Kamal, 2021	USA	Cross-sectional study	347 CD	17 tertiary referral centers	sCDAI	Patient report	50 (14)	7
Simon, 2022	France	Cross-sectional study	229CD	A hospital	HBI	Patient report; Wexner score; Vaizey score	41 (18)	9
Jiang, 2023	USA	Cross-sectional study	403 CD	National online survey	SIBDQ	Patient report; RFIS	233 (58)	9
Karki, 2023	USA	Cross-sectional study	929 CD	Multi-country online survey	SIBDQ	Patient report; RFIS	436 (47)	9
Matsumoto, 2023	Japan	Cross-sectional study	84 CD	National online survey	N/A	Patient report	37 (44)	6
Codes, 2023	Brazil	Cross-sectional study	104 CD	A referral center	HBI; PADI	Patient report; Wexner score	51 (49)	9
Ilsar, 2024	Israel	Cross-sectional study	70 CD	An IBD clinic	HBI	Patient report; FISI	60 (86)	9
Wang, 2024	China	Cohort study	600 CD	4 IBD centers	CDAI; PDAI	Patient report; Wexner incontinence score	134 (22)	8

CDAI, Crohn’s Disease Activity Index; sCDAI, Short Crohn’s Disease Activity Index; PDAI, Perianal Disease Activity Index; CCIS, Cleveland Clinic Incontinence Score; HBI, Harvey Bradshaw Index; SIBDQ, Short Inflammatory Bowel Disease Questionnaire; RFIS, Revised Fecal Incontinence Scale; FISI, Fecal Incontinence Severity Index.

#### 3.1.2 The prevalence of FI

**FIGURE 2:**
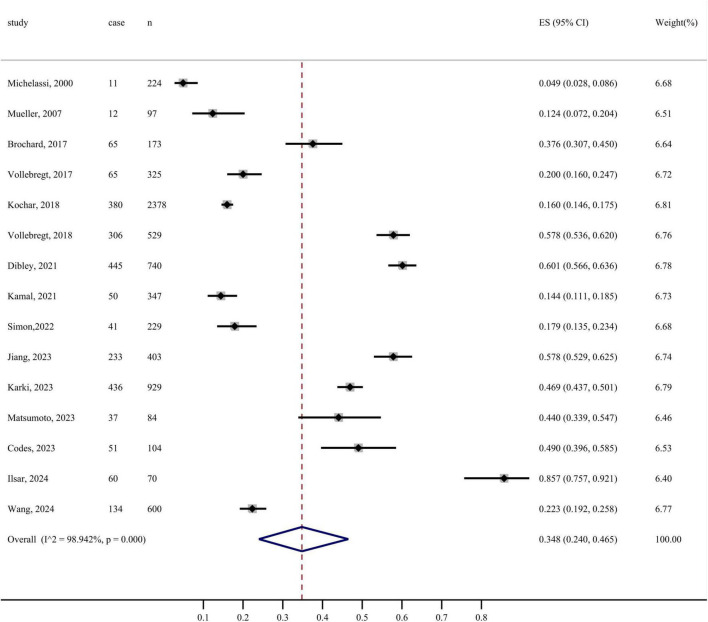
Forest plot of pooled prevalence of FI in patients with CD.

In this study, the prevalence of FI using random effects model was 34.8% (95% CI: 24.0%–46.5%, I^2^ = 98.942%, *P* < 0.001). The forest plot of the prevalence of FI is depicted in Figure 2. Sensitivity analysis was performed using random-effects models to determine the effect of individual studies on the pooled estimate. Excluding any of the studies, the combined results of the remaining studies were statistically significant, indicating the robustness of the pooled prevalence of FI among patients with CD (Supplementary material).

#### 3.1.3 Subgroup analysis

Subgroup analysis revealed that, when categorized by sample source, the prevalence of FI among patients with CD was 52.3% for the online group and 28.9% for the offline group. Stratified by study design, the incidence of FI in patients with CD was 41.7% in cross-sectional studies and 17.7% in cohort studies. Additionally, when categorized by diagnostic criteria, the prevalence of FI based on self-report alone was 24.5%, whereas the prevalence confirmed by professional instruments was 40.2% (Table 2).

**TABLE 2:** Subgroup analysis of the prevalence of FI in CD patients.

Subgroups	Number of included studies	Heterogeneity study		Prevalence
		I^2^	*P*	
**Sample source**				
Online	4	87.7%	<0.001	52.3% (45.3%, 59.3%)
Offline	11	98.7%	<0.001	28.9% (17.7%, 41.7%)
**Study type**				
Cross-sectional study	11	99.1%	<0.001	41.7% (28.0%, 56.2%)
Cohort study	4	96.3%	<0.001	17.7% (6.7%, 32.5%)
**Diagnostic criteria**				
Patient report	5	99.1%	<0.001	24.5% (4.7%, 52.9%)
Professional instrument	10	99.0%	<0.001	40.2% (27.2%, 53.9%)

#### 3.1.4 Risk factors

Seven studies (28, 29, 32–34, 37, 39) have investigated factors influencing FI in patients with CD and conducted statistical analyses to identify potential risk factors. These factors can be categorized into clinical characteristics, disease-related factors, and demographic variables. Disease-related factors encompass the Harvey Bradshaw Index (HBI) (28, 33, 37), Simplified Crohn’s Disease Activity Index (sCDAI) > 150 (32), Perianal Disease Activity Index (PDAI) > 4 (39), disease duration (28), penetrating disease behavior (39), recent CD flare-ups (34), physician global assessment (32), and strict disease control (29). Clinical characteristics associated with FI include perianal disease (29, 37, 39), prior anoperineal surgery (28, 37), diarrhea (three stools at least per day) (33), liquid stool (29, 32, 33, 37), abdominal pain (33), and fecal urgency (32). Demographic variables associated with FI include age (32, 33, 39), ethnicity (34), and the number of childbirths in women (28). These factors were found to be statistically significant predictors of FI in patients with CD (*P* < 0.05) (see the Supplementary material).

#### 3.1.5 Publication bias

According to the funnel plot, Egger’s test (*t* = 0.90, *P* = 0.386 > 0.05), suggesting that there is no publication bias in the literature of this study.

### 3.2 Pathophysiology

Six studies, including a total of 324 patients with CD and 34 healthy controls, evaluated the anal sphincter dysfunction that may lead to FI in CD patients (Table 3). Various methods such as anorectal manometry (ARM), balloon expulsion tests, and 3D endoanal ultrasound were used to assess anal sphincter function. The studies suggest that CD patients may exhibit anal sphincter dysfunction even in the absence of macroscopic perianal lesions. These dysfunctions may include damage to the anal sphincter muscles, abnormalities in rectal sensation, and disorders in defecation coordination. Overall, the results of studies describing the pathophysiology of FI in CD are contradictory.

**TABLE 3:** Summary of studies evaluating anorectal function in FI in CD patients.

Study	Country	Study type	Subjects	Source of subject recruitment	Methods to assess disease activity	Diagnostic criteria for FI	No. (%) with FI	Methods of assessing anorectal function	Quality assessment
Papathanasopoulos, 2013	Greece	Cross-sectional study	52 patients (38 CD, 14 healthy controls)	An academic tertiary-care center	CDAI	Patient report	13 (25)	1. Anorectal Manometry; 2. FRI; 3. Rectal Distension Studies; 4. Rectal Compliance; 5. EAUS	High
Litta, 2021	Italy	Cohort study	50 patients (30 CD, 20 healthy controls)	A medical center	HBI; Endoscopy; Fecal calprotectin	Patient report; CCFI	6 (12)	1. ARM; 2.3D-EAUS; 3. Endoscopy	Moderate
Portilla, 2015	Spanish	Cross-sectional study	95 CD	A hospital	3D ARU	Patient report	7 (7)	1.3D ARU	High
Albuquerque, 2021	UK	Cohort study	16 CD	A hospital	HBI; PDAI; 3D-EAUS	Patient report; Wexner’s score	4 (25)	1. HR-ARM; 2. Balloon expulsion Test; 3. 3D-EAUS	Moderate
Codes, 2023	Brazil	Cross-sectional study	104 CD	A referral center	HBI; PADI	Patient report; Wexner’s score	51 (49)	1. ARM	High
Chrysos, 2001	Greece	Cross-sectional study	41 CD	A hospital	Histologic Lesions; Endoscopy	Patient report	8 (20)	1. ARM	Moderate

FRI, fatigue rate index; EAUS, endoanal ultrasound; ARM, anorectal manometry; HR-ARM, High-Resolution Anorectal Manometry; ARU, anorectal ultrasonography.

Five studies assessed anal sphincter pressure, with three finding lower resting anal pressure in patients with FI (37, 40, 44), while two reported normal resting and squeeze pressures in all participants (41, 43). One study indicated that anal resting and squeeze pressures were not associated with the severity of FI (41). Three studies reported reduced rectal compliance in patients with FI (37, 40, 41), while one found no difference in rectal compliance between FI patients and healthy controls (44). Five studies investigated anorectal sensitivity. One study reported that FI patients exhibited both rectal hyposensitivity and hypersensitivity (43). Two studies found rectal hypersensitivity in FI patients (40, 41). Meanwhile, two other studies reported no association between FI and rectal sensitivity (37, 44). Six studies evaluated anal sphincter function, with three (41, 43, 44) using endoanal ultrasound (EAUS) and one (42) using anorectal ultrasonography (ARU) to assess the morphology and integrity of the internal and external anal sphincters, finding that sphincter defects did not necessarily lead to FI in two studies (42, 43). One study assessed the correlation between the anal fatigue rate index (FRI) and the severity of FI (41). Due to methodological differences, the results of studies could not be pooled for analysis.

### 3.3 Potential treatments

Although a variety of treatments are available for FI, there is a paucity of research specifically addressing therapies for FI in patients with CD. A rigorous selection process identified five studies that investigate potential treatment measures for FI in CD patients (Table 4). One study indicated that despite no significant change in the Wexner score following PTNS therapy, 43% (3/7) of patients reported substantial improvements in symptoms and quality of life, with subjective perceptions of improvement correlating with enhanced quality of life (45). Another study demonstrated that sacral nerve stimulation (SNS) improved FI in CD patients with damage to the internal and external anal sphincters, with 100% (5/5) of treated patients showing improved bowel control, as evidenced by enhanced Wexner scores and quality of life (46). Two studies by Khera et al. demonstrated that the majority of patients experienced significant improvements in symptoms and quality of life after undergoing pelvic floor behavior treatment when pharmacological treatments were ineffective, yet no correlation was established between the number of therapy sessions and therapeutic outcomes (47, 48). A study also identified the potential benefits of anti-TNF treatment and surgical closure in ameliorating FI symptoms in CD patients, with 28% (21/76) of patients had improved abstinence (fewer problems with FI) after treatment, and highlighted that radiological healing is associated with improved long-term efficacy (49). In summary, these studies are limited by their small sample sizes, underscoring the necessity for larger controlled trials to further substantiate the efficacy of these treatment measures.

**TABLE 4:** Summary of studies evaluating therapeutics for FI in CD patients.

Study	Country	Study Type	Subjects	Source of subject recruitment	Methods to assess disease activity	Diagnostic criteria for FI	No. (%) with FI	Treatments	*N* (%) that responded to treatments	Quality assessment
Vitton, 2009	France	Case Series Study	7 CD	Three hospitals	HBI	Patient report; Wexner’s score	7 (100)	Transcutaneous posterior tibial nerve electrical stimulation	3 (43)	High
Khera, 2022	Australia	Cohort study	13 CD	Two hospitals	Fecal calprotectin; Endoscopy; MRI	Patient report; St. Marks incontinence score	–	Pelvic floor behavioral treatment	–	Moderate
Khera, 2019	Australia	Cohort study	24 CD	A multidisciplinary clinic	HBI; Endoscopy	Patient report	12 (50)	Gut-Directed Pelvic Floor Behavioral Treatment	11 (92)	Moderate
Vitton, 2008	France	Case Series Study	5 CD	A hospital	CDAI; MRI	Patient report; Wexner’s score	5 (100)	Sacral nerve stimulation	5 (100)	High
Praag, 2023	the Netherlands	RCT	76 CD	Multiple hospitals	MRI	Patient report	76 (100)	Short-term anti-TNF therapy with surgical closure and anti-TNF therapy alone	21 (28)	High

## 4 Discussion

The aim of this systematic review and meta-analysis is to elucidate the prevalence, pathophysiology, and treatments for FI in patients with CD. The results indicate that the pooled prevalence of FI is 34.8% (95% CI: 24.0%–46.5%, I^2^ = 98.942%, *P* < 0.001). The estimated prevalence in this meta-analysis closely resembled that of previous reviews (6, 29, 50). Given the relatively high prevalence rate of 34.8%, the clinical significance of FI in CD patients is substantial, underscoring the necessity for proactive screening to enhance early detection and management.

This meta-analysis revealed a high heterogeneity in FI prevalence estimates. This heterogeneity may be attributed to a multitude of factors, including patient age, geographic region, study design, and study quality. To date, there is an absence of a standardized diagnostic criterion for FI, with some studies relying on patient self-reporting alone, which can influence the accurate assessment of the true prevalence of FI. Given the subjective nature of diagnosing FI, the diagnostic methods employed may be a principal contributor to the observed heterogeneity. Additionally, methodological heterogeneity is another critical factor that warrants consideration. The prevalence data are sourced from diverse study designs and methodological qualities, encompassing sampling methods, sample sources, sample sizes, and data collection methodologies. Subgroup analysis reveals significant heterogeneity in the prevalence of FI across different sample sources, study types, and diagnostic criteria. Online studies report a markedly higher prevalence of FI compared to offline settings, likely reflecting patients’ misconceptions about FI diagnosis and insufficient professional healthcare support. The prevalence of FI among patients with CD observed in cross-sectional studies is generally higher than that in cohort studies, which may be related to the characteristics of the study design, such as sample size and selection bias. Regarding the diagnostic criteria for FI, the prevalence based solely on patient self-report is significantly lower than that assessed with professional instruments. This discrepancy may arise from patients’ individualized understanding of the definition and severity of FI, or their reluctance to fully disclose symptoms due to social and psychological factors, such as shame and embarrassment (51, 52). Crucially, these limitations reflect a broader scarcity of high-quality studies specifically investigating FI in CD populations. The paucity of standardized diagnostic frameworks, longitudinal data on FI progression, and multinational cohorts hinders both clinical decision-making and mechanistic insights. Future research prioritizing prospectively designed studies with unified FI assessment protocols may resolve existing discrepancies, while international collaborations could address geographic disparities in healthcare access and cultural reporting biases.

Gender, age, and disease duration are widely recognized as significant factors affecting FI in patients with CD. A study has indicated that the prevalence of FI is higher in female CD patients than in males, potentially due to differences in sphincter structure and an increased risk of pelvic floor muscle and nerve damage during childbirth (53, 54). Additionally, the risk of FI increases with age, with the incidence of FI in older adults >70 years of age being about 15% (55, 56). Vollebregt found that for every additional year of age in patients, the relative risk of FI increases by 1.03 times (29). This may be related to the decline in anal sphincter function and an increase in complications with advancing age. The length of disease duration in CD patients is also a significant factor affecting anal function. Research shows that among CD patients of reproductive age, long disease duration is an independent risk factor for FI (28). As the disease duration extends, the probability of developing perianal diseases, especially anal fistulas, gradually increases, with a cumulative risk of 21% after 10 years and 26%–28% after 20 years (57, 58). The location or type of the fistula, as well as fistula surgery, significantly increase the risk of FI. Other factors such as liquid stool, perianal diseases, HBI, CDAI, fecal urgency, worse physician global assessment, and previous anoperineal surgery are also considered to be associated with FI in CD patients. The complex interplay of these factors makes it difficult to identify a single influencing factor. Therefore, although we have recognized multiple potential factors affecting FI in CD patients, the limited number of studies and methodological differences prevent effective statistical meta-analysis. Collaborative efforts are urgently needed to harmonize data protocols, particularly for capturing comprehensive demographic and clinical details. Such standardization will enable robust multivariate analyses to disentangle the interplay of biological and environmental factors driving FI. However, regional disparities may significantly influence the prevalence, presentation, and management of FI. Future research must prioritize addressing these regional differences to ensure that standardized data protocols and predictive models account for local variations. This approach is critical for advancing personalized risk prediction and targeted management in patients with CD, thereby improving health equity and clinical outcomes across diverse regions.

Research into the anorectal pathophysiology behind FI in CD patients is notably scarce, indicating a significant gap in our understanding. Existing literature suggests that even in the absence of macroscopic perianal lesions, CD patients may exhibit anorectal dysfunction, which could include damage to the anal sphincter, abnormalities in rectal sensation, and disorders in defecation coordination (40). However, the research findings in this area are often contradictory, highlighting the complexity of anorectal pathophysiology in CD patients, which has led to a lack of consensus on the definitive pathophysiological mechanisms of FI in CD. We have observed that CD patients with FI may exhibit either rectal hyposensitivity or hypersensitivity. One possible explanation for these conflicting findings lies in the heterogeneity of CD itself. The clinical course of CD is highly variable, with periods of active inflammation and remission that may significantly influence anorectal function. During periods of disease activity, rectal sensitivity is often heightened due to mucosal inflammation and perianal disease, which can result in increased urgency and a reduced threshold for stool perception (41). This heightened sensitivity may contribute to a higher incidence of fecal urgency and incontinence. Conversely, during periods of remission, particularly in cases where fibrosis and scarring have occurred, rectal sensitivity may be diminished. It is noteworthy that anorectal ultrasound and manometric studies are crucial tools in assessing these dysfunctions and can aid in tailoring management strategies. Interestingly, while the clinical assessment of anorectal function remains central, the inclusion of advanced imaging techniques, such as three-dimensional anorectal ultrasound, has shown promise in detecting subtle changes in anorectal function not always captured by clinical exams (42). This underscores the importance of combining clinical, manometric, and imaging data for a more comprehensive understanding of anorectal dysfunction in CD patients with FI. To achieve a more comprehensive understanding of the pathophysiology, future research must focus on elucidating these underlying mechanisms, which will facilitate the development of targeted and effective treatment strategies for controlling fecal incontinence in these patients. Future research should also explore the heterogeneity of CD and its impact on anorectal function, as well as the potential role of advanced imaging techniques and neuromodulation therapies in the diagnosis and treatment of anorectal dysfunction.

In exploring treatment measures for FI in patients with CD, we have found that although there are numerous methods for treating FI, research specifically targeting the CD patient population is relatively scarce, and the sample sizes of existing studies are generally small. This limitation not only restricts the generalizability of the study results but may also lead to the neglect of the heterogeneity of treatment effects. Neuromodulation has been shown to be effective for FI. SNS can improve bowel control and enhance quality of life in patients with both internal and external anal sphincter disruption. Interestingly, while PTNS has not been shown to objectively improve FI, many patients report symptom relief and improved quality of life. When pharmacological treatments are ineffective, pelvic floor behavioral therapy has also been demonstrated to improve FI; however, evidence regarding its efficacy in patients with quiescent CD remains limited. It is worth noting that psychotherapy plays an important role. Although no studies have yet demonstrated the precise effects of psychotherapy on FI in patients with CD, research has shown that psychosocial factors are significantly associated with comorbidities of IBD, including depression and anxiety (59), which may influence bowel function through complex gut-brain axis mechanisms (60). Future research could investigate the potential role of psychological treatments in improving FI and enhancing overall quality of life. Other treatments for FI, such as dietary modifications, biofeedback therapy, and implantation of an artificial bowel sphincter, have not yet been studied in CD patients. In conclusion, the treatment of FI in CD patients requires a multidisciplinary approach that combines pharmacological, behavioral, and surgical treatments. Given the methodological limitations of existing studies, large-scale, rigorously designed RCTs are urgently needed to provide robust evidence on the efficacy and safety of these interventions in CD. Future research should prioritize adequately powered, multicenter RCTs with extended follow-up periods to ensure statistical reliability and long-term outcome assessment. Additionally, such trials should incorporate standardized disease severity metrics, clinically meaningful endpoints, and comprehensive patient-reported outcomes to enhance generalizability and clinical relevance.

While this study benefits from strengths such as research selection, dual review process, data extraction, stringent inclusion criteria, and quality assessment by two independent reviewers, several limitations must be acknowledged. Despite the sample size being one of the largest collected to date, random errors are challenging to eliminate entirely. Many studies lack detailed characteristics such as age, ethnicity, geographic location, and disease subtypes, which precludes the assessment of clinically relevant factors that may influence the risk of FI in patients with CD. The number of studies on the pathophysiology and treatments of FI in patients with CD is not sufficiently to allow for meta-analysis. Furthermore, our study was limited by heterogeneity in the analysis of pooled prevalence. Given the high heterogeneity observed, the pooled prevalence estimate should be interpreted with caution.

## 5 Conclusion

This study provides insights into the prevalence, pathophysiology, and treatments of FI in patients with CD. The findings indicate that the prevalence of FI among CD patients is 34.8%, aligning with previous reports. Our research underscores the current lack of a unified standard for diagnosing FI. Recent studies have primarily focused on determining the prevalence and risk factors of FI in patients with CD and evaluating its impact on quality of life. While these studies provide preliminary insights, further research is required to elucidate the pathophysiological mechanisms of FI and to develop effective management and treatment strategies.

## Data Availability

The original contributions presented in this study are included in this article/Supplementary material, further inquiries can be directed to the corresponding author.
