# Supplementary material for: The prevalence, pathophysiology, and treatment of fecal incontinence in patients with Crohn’s disease: a systematic review and meta-analysis
Source: Front Med (Lausanne). 2025 May 27;12:1590971. doi: 10.3389/fmed.2025.1590971 (PMC12149122; doi:10.3389/fmed.2025.1590971)
Supplement: Supplementary file 1 [file Data_Sheet_1.zip › Supplementary Material Presentation/PRISMA Flowchart.docx]

**Identification of studies via databases and registers**

Records removed *before screening*:

Duplicate records removed (n =1437 )

Records identified from:

Databases (n =2703 )

PubMed (n =352 )

Embase (n =754 )

Cochrane (n =52 )

Web of Science (n =676 )

Scopus (n =869 )

**Identification**

Reports not retrieved:

Title and abstract not relevant,

(n =1090 )

Records screened

(n =1266 )

Records excluded:

Non-English studies(n =72 )

Meta (n =6 )

Review (n =21 )

Animal experiment (n =3 )

Case report, Letter, Comment, or Conference abstract (n =32 )

Reports sought for retrieval

(n =176 )

**Screening**

Reports excluded:

Unable to find full text(n=17 )

Reports assessed for eligibility

(n =42 )

Studies included in review

(n =25 )

**Included**

15 studies assessed

prevalence

5 studies assessed

therapeutic interventions

6 studies assessed

pathophysiology

Figure 1. PRISMA flowchart of included studies.

(Note: one study evaluated both prevalence and pathophysiology)

*Consider, if feasible to do so, reporting the number of records identified from each database or register searched (rather than the total number across all databases/registers).

**If automation tools were used, indicate how many records were excluded by a human and how many were excluded by automation tools.

Source: Page MJ, et al. BMJ 2021;372:n71. doi: 10.1136/bmj.n71.

This work is licensed under CC BY 4.0. To view a copy of this license, visit <https://creativecommons.org/licenses/by/4.0/>
