# Supplementary material for: The prevalence, pathophysiology, and treatment of fecal incontinence in patients with Crohn’s disease: a systematic review and meta-analysis
Source: Front Med (Lausanne). 2025 May 27;12:1590971. doi: 10.3389/fmed.2025.1590971 (PMC12149122; doi:10.3389/fmed.2025.1590971)
Supplement: Supplementary file 1 [file Data_Sheet_1.zip › Supplementary Material Presentation/Quality assessment.docx]

Table 1 The JBI Critical Appraisal Checklist for Studies Reporting Prevalence Data

| Study | Item 1 | Item 2 | Item 3 | Item 4 | Item 5 | Item 6 | Item 7 | Item 8 | Item 9 | Totals |
| --- | --- | --- | --- | --- | --- | --- | --- | --- | --- | --- |
| Michelassi, 2000 | 1 | 1 | 1 | 1 | 1 | 0 | 0 | 1 | 1 | 7 |
| Mueller, 2007 | 1 | 1 | 1 | 1 | 1 | 0 | 0 | 1 | 1 | 7 |
| Brochard, 2017 | 1 | 1 | 1 | 1 | 1 | 1 | 1 | 1 | 1 | 9 |
| Vollebregt, 2017 | 1 | 1 | 1 | 1 | 1 | 1 | 1 | 1 | 1 | 9 |
| Kochar, 2018 | 1 | 1 | 1 | 1 | 1 | 1 | 1 | 1 | 1 | 9 |
| Vollebregt, 2018 | 1 | 1 | 1 | 1 | 1 | 0 | 0 | 1 | 1 | 7 |
| Dibley, 2021 | 1 | 1 | 1 | 1 | 1 | 0 | 0 | 1 | 1 | 7 |
| Kamal, 2021 | 1 | 1 | 1 | 1 | 1 | 0 | 0 | 1 | 1 | 7 |
| Simon, 2022 | 1 | 1 | 1 | 1 | 1 | 1 | 1 | 1 | 1 | 9 |
| Jiang, 2023 | 1 | 1 | 1 | 1 | 1 | 1 | 1 | 1 | 1 | 9 |
| Karki, 2023 | 1 | 1 | 1 | 1 | 1 | 1 | 1 | 1 | 1 | 9 |
| Matsumoto, 2023 | 1 | 1 | 1 | 1 | 1 | 0 | 0 | 1 | 0 | 6 |
| Codes, 2023 | 1 | 1 | 1 | 1 | 1 | 1 | 1 | 1 | 1 | 9 |
| Ilsar, 2024 | 1 | 1 | 1 | 1 | 1 | 1 | 1 | 1 | 1 | 9 |
| Wang, 2024 | 1 | 1 | 1 | 1 | 1 | 0 | 1 | 1 | 1 | 8 |

Note:

1. Was the sample frame appropriate to address the target population?
2. Were study participants sampled in an appropriate way?
3. Was the sample size adequate?
4. Were the study subjects and the setting described in detail?
5. Was the data analysis conducted with sufficient coverage of the identified sample?
6. Were valid methods used for the identification of the condition?
7. Was the condition measured in a standard, reliable way for all participants?
8. Was there appropriate statistical analysis?
9. Was the response rate adequate, and if not, was the low response rate managed appropriately?

Table 2 Quality assessment of cross-sectional studies

| Study | Item 1 | Item 2 | Item 3 | Item 4 | Item 5 | Item 6 | Item 7 | Item 8 | Item 9 | Item 10 | Item 11 | Totals | Grade |
| --- | --- | --- | --- | --- | --- | --- | --- | --- | --- | --- | --- | --- | --- |
| Papathanasopoulos, 2013 | 1 | 1 | 1 | 1 | 0 | 1 | 1 | 1 | 0 | 1 | 0 | 8 | High |
| Codes, 2023 | 1 | 1 | 1 | 1 | 1 | 1 | 1 | 1 | 0 | 1 | 0 | 9 | High |
| Portilla,2015 | 1 | 1 | 1 | 1 | 0 | 1 | 1 | 1 | 0 | 1 | 0 | 8 | High |
| Chrysos, 2001 | 1 | 1 | 0 | 1 | 0 | 1 | 1 | 1 | 0 | 1 | 0 | 7 | Medium |

Note:

1) Define the source of information (survey, record review)

2) List inclusion and exclusion criteria for exposed and unexposed subjects (cases and controls) or refer to previous publications

3) Indicate time period used for identifying patients

4) Indicate whether or not subjects were consecutive if not population-based

5) Indicate if evaluators of subjective components of study were masked to other aspects of the status of the participants

6) Describe any assessments undertaken for quality assurance purposes (e.g., test/retest of primary outcome measurements)

7) Explain any patient exclusions from analysis

8) Describe how confounding was assessed and/or controlled.

9) If applicable, explain how missing data were handled in the analysis

10) Summarize patient response rates and completeness of data collection

11) Clarify what follow-up, if any, was expected and the percentage of patients for which incomplete data or follow-up was obtained

Table 3 Quality assessment of cohort studies

| Study | Selection | | | | Comparability | Outcome | | | Totals | Grade |
| --- | --- | --- | --- | --- | --- | --- | --- | --- | --- | --- |
|  | Item 1 | Item 2 | Item 3 | Item 4 |  | Item 1 | Item 2 | Item 3 |  |  |
| Albuquerque, 2021 | 1 | 0 | 1 | 1 | 0 | 1 | 0 | 0 | 4 | Medium |
| Litta,2021 | 1 | 0 | 0 | 1 | 2 | 1 | 0 | 0 | 5 | Medium |
| Khera,2022 | 1 | 0 | 1 | 1 | 2 | 1 | 0 | 0 | 6 | Medium |
| Khera,2019 | 1 | 0 | 1 | 1 | 2 | 1 | 0 | 0 | 6 | Medium |

Note:

Selection 1) Representativeness of the exposed cohort a) Truly representative (one star) b) Somewhat representative (one star) c) Selected group d) No description of the derivation of the cohort 2) Selection of the non-exposed cohort a) Drawn from the same community as the exposed cohort (one star) b) Drawn from a different source c) No description of the derivation of the non exposed cohort 3) Ascertainment of exposure a) Secure record (e.g., surgical record) (one star) b) Structured interview (one star) c) Written self report d) No description e) Other 4) Demonstration that outcome of interest was not present at start of study a) Yes (one star) b) No

Comparability 1) Comparability of cohorts on the basis of the design or analysis controlled for confounders a) The study controls for age, sex and marital status (one star) b) Study controls for other factors (list) (one star) c) Cohorts are not comparable on the basis of the design or analysis controlled for confounders

Outcome 1) Assessment of outcome a) Independent blind assessment (one star) b) Record linkage (one star) c) Self report d) No description e) Other 2) Was follow-up long enough for outcomes to occur a) Yes (one star) b) No 3) Adequacy of follow-up of cohorts a) Complete follow up- all subject accounted for (one star) b) Subjects lost to follow up unlikely to introduce bias- number lost less than or equal to 20% or description of those lost suggested no different from those followed. (one star) c) Follow up rate less than 80% and no description of those lost d) No statement

Table 4 Quality assessment of case series studies

| Study | Item 1 | Item 2 | Item 3 | Item 4 | Item 5 | Item 6 | Item 7 | Item 8 | Item 9 | Item 10 | Totals | Grade |
| --- | --- | --- | --- | --- | --- | --- | --- | --- | --- | --- | --- | --- |
| Vitton, 2009 | 1 | 1 | 1 | 0 | 1 | 1 | 1 | 1 | 1 | 1 | 9 | High |
| Vitton, 2008 | 1 | 1 | 1 | 0 | 1 | 1 | 1 | 1 | 1 | 1 | 9 | High |

Note:

1)Were there clear criteria for inclusion in the case series?

2)Was the condition measured in a standard, reliable way for all participants included in the case series?

3)Were valid methods used for identification of the condition for all participants included in the case series?

4)Did the case series have consecutive inclusion of participants?

5)Did the case series have complete inclusion of participants?

6)Was there clear reporting of the demographics of the participants in the study?

7)Was there clear reporting of clinical information of the participants?

8)Were the outcomes or follow-up results of cases clearly reported?

9)Was there clear reporting of the presenting sites'/clinics' demographic information?

10)Was statistical analysis appropriate?

Table 5 Quality assessment of RCT

| Study ID | Randomization process | Deviations from intended interventions | Missing outcome data | Measurement of the outcome | Selection of the reported result | Overall |
| --- | --- | --- | --- | --- | --- | --- |
| Praag 2023 | 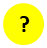 | 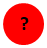 | 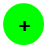 | 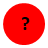 | 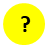 | 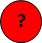 |


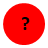

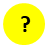

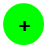


Low risk Some concerns High risk
