# Supplementary material for: The prevalence, pathophysiology, and treatment of fecal incontinence in patients with Crohn’s disease: a systematic review and meta-analysis
Source: Front Med (Lausanne). 2025 May 27;12:1590971. doi: 10.3389/fmed.2025.1590971 (PMC12149122; doi:10.3389/fmed.2025.1590971)
Supplement: Supplementary file 1 [file Data_Sheet_1.zip › Supplementary Material Presentation/Search strategy.docx]

Table 1 PubMed Search Strategy

| ID | Search Terms | Results |
| --- | --- | --- |
| #1 | (("Crohn Disease"[Mesh]) OR (Crohn Disease[Title/Abstract])) OR (crohn*[Title/Abstract]) | 69505 |
| #2 | ((((((((((((((((((((("Fecal Incontinence"[Mesh]) OR (fecal incontinence[Title/Abstract])) OR (faecal incontinence[Title/Abstract])) OR (anal incontinence[Title/Abstract])) OR (anus incontinence[Title/Abstract])) OR (bowel incontinence[Title/Abstract])) OR (feces incontinence[Title/Abstract])) OR (feces incontinence device[Title/Abstract])) OR (fecal soiling[Title/Abstract])) OR (defecation incontinence[Title/Abstract])) OR (encopresia[Title/Abstract])) OR (encopresis[Title/Abstract])) OR (encopresy[Title/Abstract])) OR (excretory incontinence[Title/Abstract])) OR (faeces incontinence[Title/Abstract])) OR (fecal incontinency[Title/Abstract])) OR (incontinence, anal[Title/Abstract])) OR (incontinentia alvi[Title/Abstract])) OR (intestinal incontinence[Title/Abstract])) OR (rectal incontinence[Title/Abstract])) OR (rectum incontinence[Title/Abstract])) OR (stool incontinence[Title/Abstract]) | 16754 |
| #3 | ((("Crohn Disease"[Mesh]) OR (Crohn Disease[Title/Abstract])) OR (crohn*[Title/Abstract])) AND (((((((((((((((((((((("Fecal Incontinence"[Mesh]) OR (fecal incontinence[Title/Abstract])) OR (faecal incontinence[Title/Abstract])) OR (anal incontinence[Title/Abstract])) OR (anus incontinence[Title/Abstract])) OR (bowel incontinence[Title/Abstract])) OR (feces incontinence[Title/Abstract])) OR (feces incontinence device[Title/Abstract])) OR (fecal soiling[Title/Abstract])) OR (defecation incontinence[Title/Abstract])) OR (encopresia[Title/Abstract])) OR (encopresis[Title/Abstract])) OR (encopresy[Title/Abstract])) OR (excretory incontinence[Title/Abstract])) OR (faeces incontinence[Title/Abstract])) OR (fecal incontinency[Title/Abstract])) OR (incontinence, anal[Title/Abstract])) OR (incontinentia alvi[Title/Abstract])) OR (intestinal incontinence[Title/Abstract])) OR (rectal incontinence[Title/Abstract])) OR (rectum incontinence[Title/Abstract])) OR (stool incontinence[Title/Abstract])) | 352 |

Table 2 Web of Science Search Strategy

| ID | Search Terms | Results |
| --- | --- | --- |
| #1 | (TS=(Crohn Disease) OR TS=(crohn*)) | 121597 |
| #2 | (TS=(fecal incontinence) OR TS=(faecal incontinence) OR TS=(anal incontinence) OR TS=(anus incontinence) OR TS=(bowel incontinence) OR TS=(feces incontinence) OR TS=(feces incontinence device) OR TS=(fecal soiling) OR TS=(defecation incontinence) OR TS=(encopresia) OR TS=(encopresis) OR TS=(encopresy) OR TS=(excretory incontinence) OR TS=(faeces incontinence) OR TS=(fecal incontinency) OR TS=(incontinence, anal) OR TS=(incontinentia alvi) OR TS=(intestinal incontinence) OR TS=(rectal incontinence) OR TS=(rectum incontinence) OR TS=(stool incontinence) OR TS=(feces incontinence)) | 45097 |
| #3 | #1 AND #2 | 676 |

Table 3 The Cochrane Library Search Strategy

| ID | Search Terms | Results |
| --- | --- | --- |
| #1 | MeSH descriptor: [Crohn Disease] explode all trees | 2307 |
| #2 | (Crohn Disease):ti,ab,kw OR (crohn*):ti,ab,kw | 6296 |
| #3 | #1 OR #2 | 6296 |
| #4 | MeSH descriptor: [Fecal Incontinence] explode all trees | 700 |
| #5 | (fecal incontinence):ti,ab,kw OR (faecal incontinence):ti,ab,kw OR (anal incontinence):ti,ab,kw OR (anus incontinence):ti,ab,kw OR (bowel incontinence):ti,ab,kw | 2755 |
| #6 | (feces incontinence):ti,ab,kw OR (feces incontinence device):ti,ab,kw OR (fecal soiling):ti,ab,kw OR (defecation incontinence):ti,ab,kw OR (encopresia):ti,ab,kw | 1336 |
| #7 | (encopresis):ti,ab,kw OR (encopresy):ti,ab,kw OR (excretory incontinence):ti,ab,kw OR (faeces incontinence):ti,ab,kw OR (fecal incontinency):ti,ab,kw | 1033 |
| #8 | (incontinence, anal):ti,ab,kw OR (incontinentia alvi):ti,ab,kw OR (intestinal incontinence):ti,ab,kw OR (rectal incontinence):ti,ab,kw OR (rectum incontinence):ti,ab,kw | 1899 |
| #9 | (stool incontinence):ti,ab,kw | 467 |
| #10 | #4 OR #5 OR #6 OR #7 OR #8 OR #9 | 3220 |
| #11 | #3 AND #10 | 52 |

Table 4 Embase Search Strategy

| ID | Search Terms | Results |
| --- | --- | --- |
| #1 | 'crohn disease'/exp | 121100 |
| #2 | 'crohn disease':ab,ti OR crohn*:ab,ti | 104186 |
| #3 | #1 OR #2 | 134379 |
| #4 | 'feces incontinence'/exp | 26023 |
| #5 | 'anal incontinence':ab,ti OR 'anus incontinence':ab,ti OR 'bowel incontinence':ab,ti OR 'defecation incontinence':ab,ti OR encopresia:ab,ti OR encopresis:ab,ti OR encopresy:ab,ti OR 'excretory incontinence':ab,ti OR 'faecal incontinence':ab,ti OR 'faeces incontinence':ab,ti OR 'fecal incontinence':ab,ti OR 'fecal incontinency':ab,ti OR 'incontinence, anal':ab,ti OR 'incontinentia alvi':ab,ti OR 'intestinal incontinence':ab,ti OR 'rectal incontinence':ab,ti OR 'rectum incontinence':ab,ti OR 'stool incontinence':ab,ti OR 'feces incontinence':ab,ti | 17339 |
| #6 | #4 OR #5 | 28495 |
| #7 | #3 AND #6 | 754 |

Table 5 Scopus Search Strategy

| ID | Search Terms | Results |
| --- | --- | --- |
| #1 | (TITLE-ABS-KEY(Crohn Disease) OR TITLE-ABS-KEY(crohn*)) | 101844 |
| #2 | (TITLE-ABS-KEY(fecal incontinence) OR TITLE-ABS-KEY(faecal incontinence) OR TITLE-ABS-KEY(anal incontinence) OR TITLE-ABS-KEY(anus incontinence) OR TITLE-ABS-KEY(bowel incontinence) OR TITLE-ABS-KEY(feces incontinence) OR TITLE-ABS-KEY(feces incontinence device) OR TITLE-ABS-KEY(fecal soiling) OR TITLE-ABS-KEY(defecation incontinence) OR TITLE-ABS-KEY(encopresia) OR TITLE-ABS-KEY(encopresis) OR TITLE-ABS-KEY(encopresy) OR TITLE-ABS-KEY(excretory incontinence) OR TITLE-ABS-KEY(faeces incontinence) OR TITLE-ABS-KEY(fecal incontinency) OR TITLE-ABS-KEY(incontinence, anal) OR TITLE-ABS-KEY(incontinentia alvi) OR TITLE-ABS-KEY(intestinal incontinence) OR TITLE-ABS-KEY(rectal incontinence) OR TITLE-ABS-KEY(rectum incontinence) OR TITLE-ABS-KEY(stool incontinence)) | 34606 |
| #3 | ((TITLE-ABS-KEY(Crohn Disease) OR TITLE-ABS-KEY(crohn*))) AND ((TITLE-ABS-KEY(fecal incontinence) OR TITLE-ABS-KEY(faecal incontinence) OR TITLE-ABS-KEY(anal incontinence) OR TITLE-ABS-KEY(anus incontinence) OR TITLE-ABS-KEY(bowel incontinence) OR TITLE-ABS-KEY(feces incontinence) OR TITLE-ABS-KEY(feces incontinence device) OR TITLE-ABS-KEY(fecal soiling) OR TITLE-ABS-KEY(defecation incontinence) OR TITLE-ABS-KEY(encopresia) OR TITLE-ABS-KEY(encopresis) OR TITLE-ABS-KEY(encopresy) OR TITLE-ABS-KEY(excretory incontinence) OR TITLE-ABS-KEY(faeces incontinence) OR TITLE-ABS-KEY(fecal incontinency) OR TITLE-ABS-KEY(incontinence, anal) OR TITLE-ABS-KEY(incontinentia alvi) OR TITLE-ABS-KEY(intestinal incontinence) OR TITLE-ABS-KEY(rectal incontinence) OR TITLE-ABS-KEY(rectum incontinence) OR TITLE-ABS-KEY(stool incontinence))) | 869 |
