# Supplementary material for: The prevalence, pathophysiology, and treatment of fecal incontinence in patients with Crohn’s disease: a systematic review and meta-analysis
Source: Front Med (Lausanne). 2025 May 27;12:1590971. doi: 10.3389/fmed.2025.1590971 (PMC12149122; doi:10.3389/fmed.2025.1590971)
Supplement: Supplementary file 1 [file Data_Sheet_1.zip › Supplementary Material Presentation/Table Risk Factors for FI in CD Patients.docx]

Table Risk Factors for FI in CD Patients

| Factor | Associated Studies | Statistical Significance (P <0.05) |
| --- | --- | --- |
| Disease-Related Factors | | |
| Harvey Bradshaw Index (HBI) | [27, 37] | Yes |
| sCDAI >150 | [32] | Yes |
| PDAI >4 | [39] | Yes |
| Disease duration | [27] | Yes |
| Penetrating disease behavior | [39] | Yes |
| Recent CD flare-ups | [34] | Yes |
| Physician global assessment | [32] | Yes |
| Strict disease control | [28] | Yes |
| Clinical Characteristics | | |
| Perianal diseases | [28, 37, 39] | Yes |
| Prior anoperineal surgery | [27, 37] | Yes |
| Liquid stool | [28, 32, 33, 37] | Yes |
| Diarrhea (three stools at least per day) | [33] | Yes |
| Abdominal pain | [33] | Yes |
| Fecal urgency | [32] | Yes |
| Demographic Variables | | |
| Age | [29, 32, 33, 39] | Yes |
| Ethnicity | [34] | Yes |
| Number of childbirths (women) | [27] | Yes |
