# Supplementary figures and images for: The prevalence, pathophysiology, and treatment of fecal incontinence in patients with Crohn’s disease: a systematic review and meta-analysis
Source: Front Med (Lausanne). 2025 May 27;12:1590971. doi: 10.3389/fmed.2025.1590971 (PMC12149122; doi:10.3389/fmed.2025.1590971)

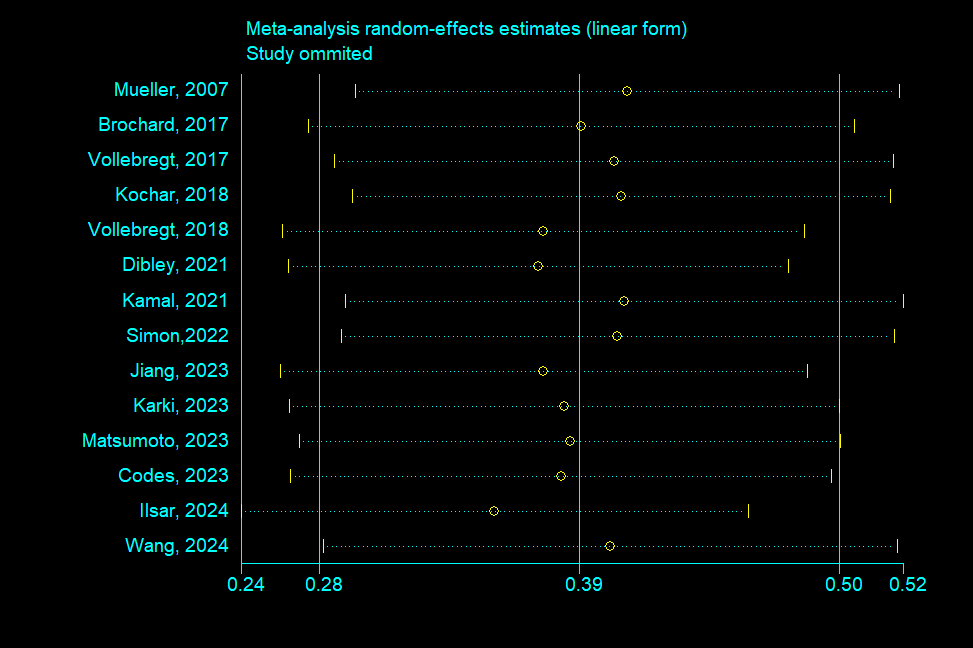

Supplement: Supplementary file 1 [file Data_Sheet_1.zip › Supplementary Material Presentation/Sensitivity analysis of the prevalence of FI in patients with CD.tif]

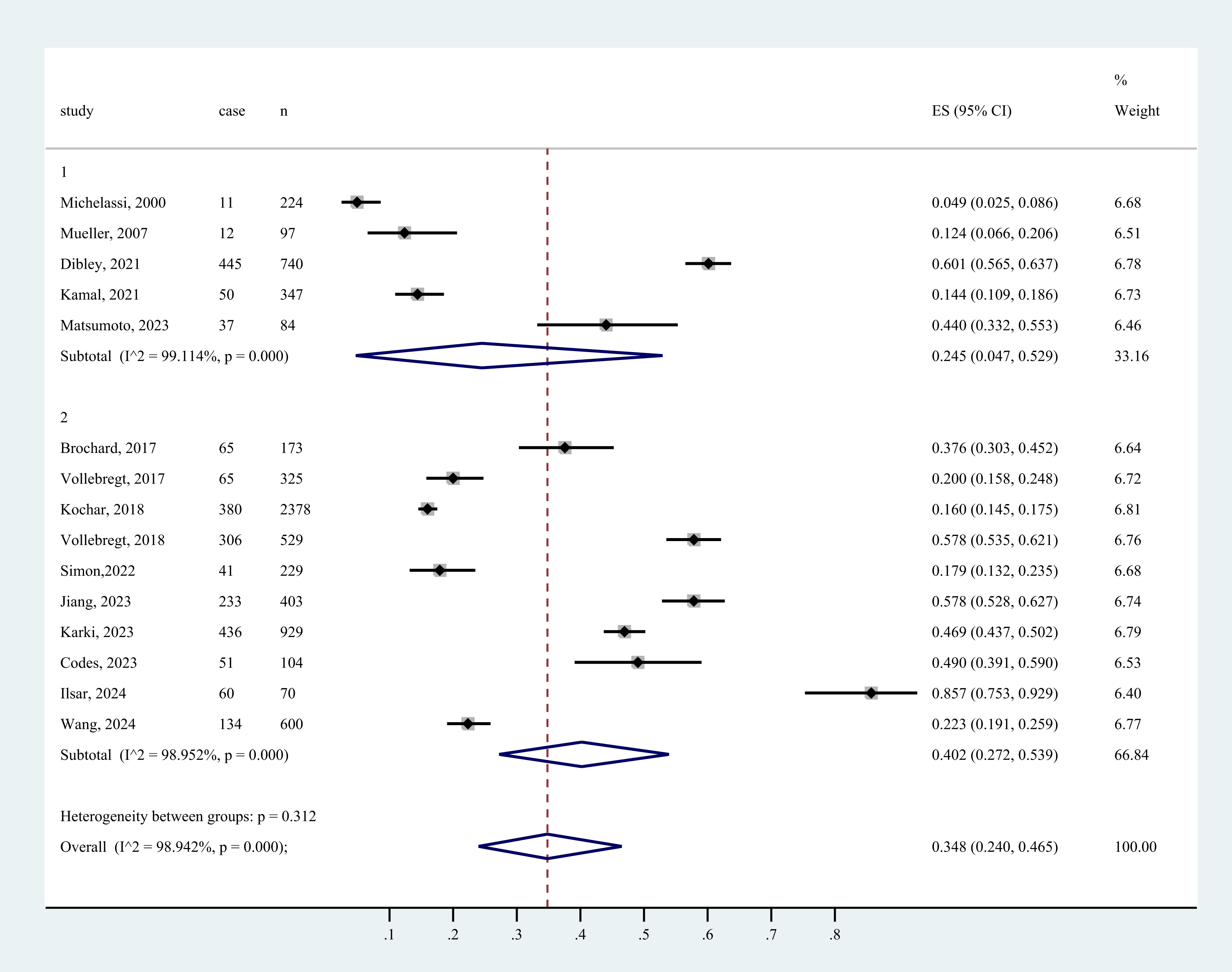

Supplement: Supplementary file 1 [file Data_Sheet_1.zip › Supplementary Material Presentation/Subgroups-diagnostic criteria.tif]

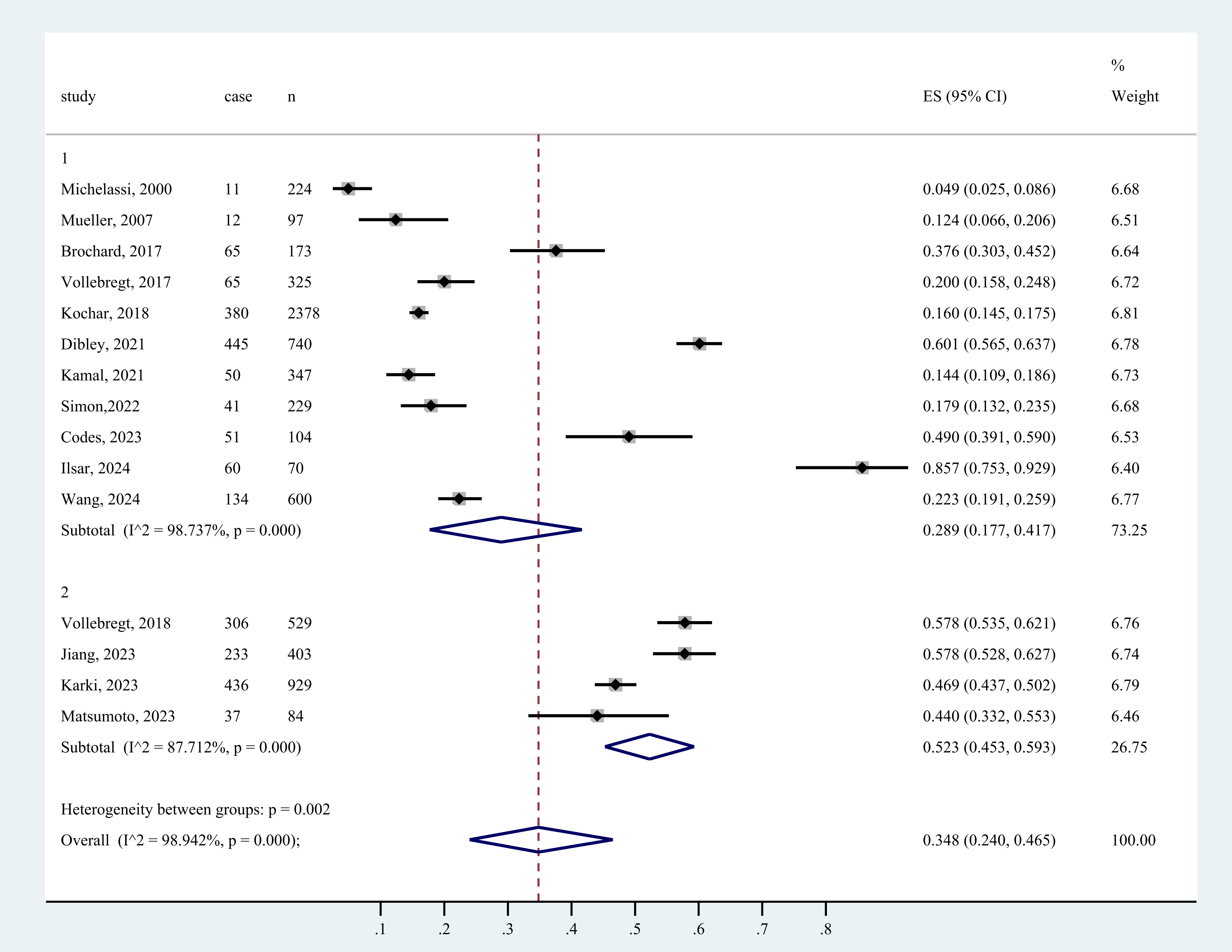

Supplement: Supplementary file 1 [file Data_Sheet_1.zip › Supplementary Material Presentation/Subgroups-sample source.tif]

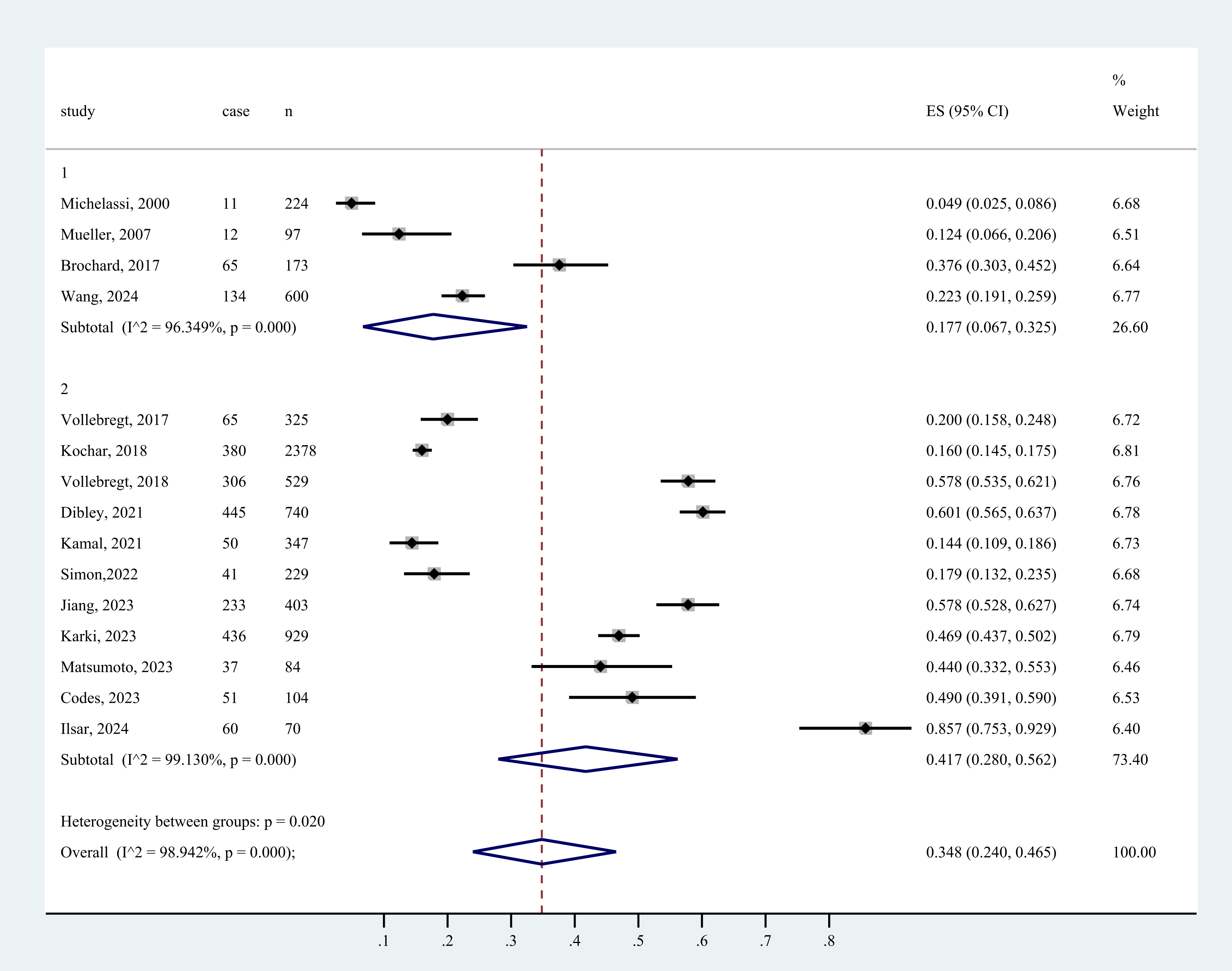

Supplement: Supplementary file 1 [file Data_Sheet_1.zip › Supplementary Material Presentation/Subgroups-study type.tif]

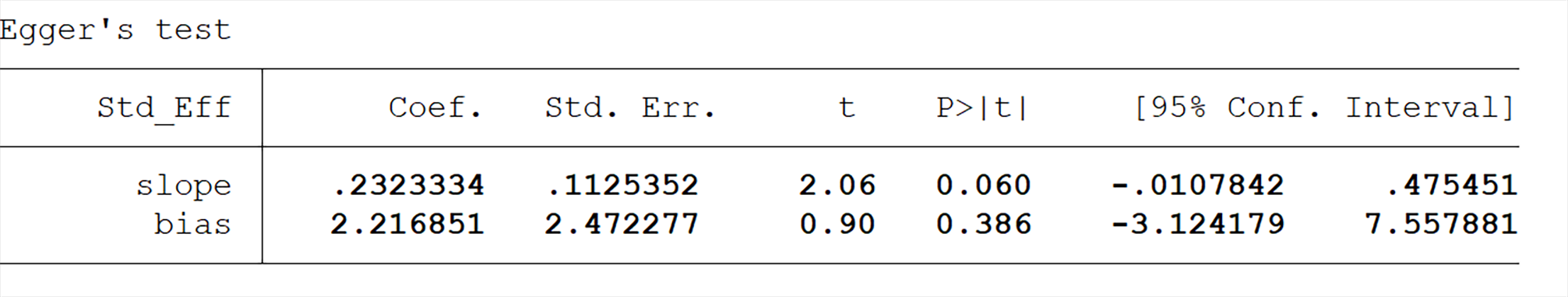

Supplement: Supplementary file 1 [file Data_Sheet_1.zip › Supplementary Material Presentation/Egger's test.tif]
